# Supplementary material for: Launching a saliva-based SARS-CoV-2 surveillance testing program on a university campus
Source: PLoS One. 2021 May 26;16(5):e0251296. doi: 10.1371/journal.pone.0251296 (PMC8153421; doi:10.1371/journal.pone.0251296)
Supplement: S1 Appendix — (PDF) [file pone.0251296.s001.pdf]

S1 Appendix
IGI FAST Exit Survey

Introduction
Thank you for taking the time to complete this survey. Your responses will help IGI to better understand and advocate for effective asymptomatic testing at UC Berkeley, and will contribute to scientific understanding of public health measures.
This should take 10-15 minutes. You may skip any questions you do not wish to answer.

Informed consent
Introduction and Purpose
The IGI FAST (Free Asymptomatic Saliva Test) Study is an investigation into preventative measures being taken to protect on-campus employees during the COVID-19 pandemic. The study's Principal Investigators are Professor Jennifer Doudna, PhD (Executive Director, Innovative Genomics Institute) and Dr. Guy Nicolette, MD (Assistant Vice Chancellor for University Health Services), and the study coordinator is Alexander Ehrenberg (PhD Student, Dept. of Integrative Biology). The purpose of this study is to model and evaluate the efficacy of asymptomatic testing to prevent the spread of COVID-19 within the campus population. Here, we ask you to complete an exit survey so we can learn more about preferences and attitudes surrounding COVID-19 testing at UC Berkeley.
Procedures
The survey, administered through Qualtrics, will involve questions about your involvement in IGI FAST, attitudes and perceptions about COVID-19, and attitudes and perceptions about COVID-10 testing. It should take about 10-15 minutes to complete. Your identity will not be recorded and data collected through this survey will not be tied to other data collected in the IGI FAST study or samples you gave through the IGI FAST Study.
Benefits
There is no direct benefit to you from taking part in this study. It is hoped that the research will inform on future COVID-19 testing strategies.
Risks/Discomforts
Some questions may make you upset or uncomfortable. You are welcome to skip any questions as you wish and may stop the survey at any time. Although every reasonable effort has been taken, confidentiality during actual Internet communication procedures cannot be guaranteed.
Confidentiality
Your study data will be handled as confidentially as possible. If results of this study are published or presented, individual names and other personally identifiable information will not be disclosed. To minimize the risks to confidentiality, we will not be collecting any identifiable information in this survey. At the conclusion of the survey period (10/30/2020), data will be downloaded from Qualtrics, stored within a UC Berkeley Box folder, and deleted from Qualtrics. When the research is completed, the data will be saved for possible use in future research done by IGI or others. We will retain these records indefinitely. The same measures described above will be taken to protect confidentiality of this study data. Your personal information may be released if required by law. Authorized representatives from the University of California may review your research data for purposes such as monitoring or managing the conduct of this study.
Compensation
You will not be paid for taking part in this survey.
Rights
Participation in research is completely voluntary. You are free to decline to take part in the project. You can decline to answer any questions and are free to stop taking part in the project

at any time. Whether or not you choose to participate, to answer any particular question, or continue participating in the project, there will be no penalty to you or loss of benefits to which you are otherwise entitled.

Questions

If you have any questions about this research, please feel free to contact [REDACTED] at [REDACTED]. If you have any questions about your rights or treatment as a research participant in this study, please contact the University of California at Berkeley's Committee for Protection of Human Subjects at [REDACTED], or e-mail [REDACTED]. **If you agree to take part in the research, please print a copy of this page and click "Accept" below.**

CPHS # [REDACTED]

Q3.1 To which age category do you belong?

- ○ 18-24 years old (1)
- ○ 25-34 years old (2)
- ○ 35-44 years old (3)
- ○ 45-54 years old (4)
- ○ 55-64 years old (5)
- ○ 65+ years old (6)

Q3.2 Which best describes your gender identity?

- ○ Man (1)
- ○ Woman (2)
- ○ Non-binary (3)
- ○ Other (7)
- ○ Prefer not to say (4)

Q3.3 Do you consider yourself to be or have origins associated with any of the following? (select all that apply)

- ☐ American Indian or Alaska Native (1)
- ☐ Asian Indian (5)
- ☐ Black or African American (2)
- ☐ Chinese (9)
- ☐ Cuban (18)
- ☐ Filipino (10)
- ☐ Guamanian or Chamorro (14)
- ☐ Japanese (11)
- ☐ Korean (12)
- ☐ Mexican, Mexican American, or Chicana/Chicano (16)
- ☐ Native Hawaiian (4)
- ☐ Puerto Rican (17)
- ☐ Samoan (15)
- ☐ Vietnamese (13)
- ☐ White (6)
- ☐ Other Latina/Latino or Spanish origin (19)
- ☐ Other Pacific Islander (7)
- ☐ Other (20)
- ☐ Prefer not to say (8)

Q3.4 What best describes your role at UC Berkeley? (Select all that apply)

- ☐ Undergraduate student (1)
- ☐ Graduate/professional student (2)
- ☐ Postdoc (10)
- ☐ Academic faculty or staff (9)
- ☐ Non-academic staff (4)

Display Question Q3.5:
If Q3.4 = Undergraduate student
Or Q3.4 = Graduate/professional student

Q3.5 Do any of the following further describe your role at UC Berkeley? (Select all that apply)

- ☐ Student researcher (1)
- ☐ Student employee (4)
- ☐ Student-athlete (active NCAA roster) (2)

Display Question Q3.6:
If Q3.4 = Postdoc
Or Q3.4 = Academic faculty or staff
Or Q3.4 = Non-academic staff
Or Q3.5 = Student researcher
Or Q3.5 = Student employee

Q3.6 Which of the following categories describe your work at UC Berkeley? (Select all that apply)

- ☐ Laboratory work (1)
- ☐ Non-laboratory research work (2)
- ☐ OLAC animal facilities staff (7)
- ☐ EH&S staff (8)
- ☐ Administrative or programmatic work (9)
- ☐ Service work (e.g. dining, retail) (10)
- ☐ Resident assistant (RA) or other residential life work (11)
- ☐ Healthcare work (e.g. UHS staff, Optometry student/resident, COVID-19 testing site worker) (3)
- ☐ Clinical laboratory work (e.g. IGI testing lab) (4)
- ☐ Custodial, maintenance, or facilities work (5)
- ☐ Student services work (12)
- ☐ Athletics/training staff (13)
- ☐ Childcare work (e.g. ECEP) (14)
- ☐ Security or UCPD work (15)
- ☐ Other (6)

Display Question 3.7:
If Q3.6 = Healthcare work (e.g. UHS staff, Optometry student/resident, COVID-19 testing site worker)

Q3.7 Do any of the following describe your work in healthcare? (Select all that apply)

▢ Patient-facing (1)

▢ Interacting (within 6' for more than 15 minutes) with patients (3)

▢ Telemedicine (4)

▢ COVID-19 clinician-administered testing (e.g. nasopharyngeal testing) (5)

▢ COVID-19 self-administered testing (e.g. monitoring IGI FAST, monitoring self-nasal swabbing) (8)

▢ Medical/optometry student (6)

▢ None of the above describe my work in healthcare (9)

Display Question 3.8:
If Q3.6 = Other

Q3.8 What else describes your work at UC Berkeley?

____

Q4.1 The following are questions about your living and working activities and conditions.

Q4.2 How frequently do you work (on or off the UC Berkeley campus) or have other responsibilities outside of your home?

○ None (1)

○ 1-2 days/week (2)

○ 3-5 days/week (3)

○ 6-7 days/week (4)

Q4.3 Are you approved to work on the UC Berkeley campus, Lawrence Berkeley National Laboratory, or in UC Berkeley-owned buildings?

- ◯ Yes (1)
- ◯ No (2)
- ◯ Not sure (4)

Display Question Q4.4:
If Q4.3 = Yes

Q4.4 In the past month, how many days a week have you typically come to campus or a campus-owned building?

- ◯ less than 1 day (5)
- ◯ 1 day (1)
- ◯ 2-3 days (2)
- ◯ 4-5 days (3)
- ◯ 6-7 days (4)

Q4.5 During the time you were participating in IGI FAST, have you worked or volunteered **off campus** in any of the following areas? (Select all that apply)

- ☐ Food service industry (1)
- ☐ In-person retail or grocery stores (6)
- ☐ Healthcare settings or nursing homes (2)
- ☐ Transportation (including 'gig economy' Uber/Lyft and food delivery) (3)
- ☐ Day cares or schools (4)
- ☐ In-home care at someone else's home (e.g. nannying, elder care) (7)
- ☐ None of the above (5)

Q4.6 Do you know anyone personally who has tested positive for COVID-19?

- ◯ Yes (1)
- ◯ No (2)
- ◯ Not sure (3)

Q4.7 What best describes your primary living situation?

- ◯ UC Berkeley owned dormitory (1)
- ◯ UC Berkeley owned apartment (including International House and University Village) (2)
- ◯ Greek housing (3)
- ◯ Co-operative housing (4)
- ◯ Apartment (5)
- ◯ House (6)
- ◯ Couch-surfing (7)
- ◯ Experiencing homelessness (8)
- ◯ Prefer to not say (9)

Display Question Q4.8:
If Q4.7 = Apartment
Or Q4.7 = House

Q4.8 How many people do you live with (total in your household or apartment unit)?

- 0 (1)
- 1 (2)
- 2-3 (3)
- 4-5 (4)
- 6-10 (5)
- 11-15 (6)
- >15 (7)

Q4.9 With how many people do you share a bedroom?

- 0 (1)
- 1 (2)
- 2-3 (3)
- 4-5 (4)
- >5 (5)

Q4.10 With how many people do you share a bathroom at your primary residence?

- 0 (1)
- 1 (2)
- 2-3 (3)
- 4-5 (4)
- >5 (5)

Display Question Q4.11:
If Q4.6 = Yes

Q4.11 Do you live with anyone who has tested positive for COVID-19 while you were living together?

- Yes (1)
- No (2)
- Not sure (3)

Q4.12 Besides those you live with, how many different people do you interact with in a typical week? Assume "interacting" involves being within 6 feet for more than 10 minutes, with or without a mask.

- 0 (1)
- 1-2 (2)
- 3-5 (3)
- 6-10 (4)
- 11-15 (5)
- >15 (6)

Q4.13 How often do you wear a mask when you are around other people that do not live with you? Assume "around" refers to being in the same room or open indoor space such as a grocery store, or within 30 feet of someone outside.

- Never (1)
- Sometimes (2)
- Most of the time (7)
- All the time (10)

Display Question Q4.14:
If Q4.13 != All the time

Q4.14 In what kind of settings would you NOT wear a mask when being around other people that do not live with you?

- ☐ When I'm outdoors (1)
- ☐ When I'm eating outdoors at a restaurant (2)
- ☐ When I'm eating indoors at a restaurant (7)
- ☐ When I'm exercising (5)
- ☐ When I don't expect to be within 6 feet of someone else (4)
- ☐ When I don't expect to be within 30 feet of someone else (3)
- ☐ Other (6)

Display Question Q4.15:
If Q4.14 = Other

Q4.15 Please describe the other conditions in which you would not wear a mask when around people that do not live with you.

____

____

____

____

____

Q4.16 How at-risk do you think you are for COVID-19 exposure due to the following? (slide the gray bar)

	No risk	High risk
	0 10 20 30 40 50 60 70 80 90 100	
Your living situation or roommates (1)		
Going to your work (2)		
Your social behavior (3)		
Travel (5)		
Shopping (6)		
Other activities (4)		

Q4.17 Do any of the following apply to you? (Select all that apply)

- ☐ I live with or care for someone over the age of 65 (1)
- ☐ I live with or care for children going to school/childcare (2)
- ☐ I live with someone who works or volunteers in the food service industry (3)
- ☐ I live with someone who works in in-person retail or grocery stores (4)
- ☐ I live with someone who works or volunteers in healthcare settings or nursing homes (5)
- ☐ I live with someone who works in transportation (including 'gig economy' Uber/Lyft and food delivery) (6)
- ☐ I live with someone who works in day cares or schools (7)
- ☐ I live with someone who works or volunteers in at-home care at another home (e.g. nannying, elder care) (8)
- ☐ I live with or care for someone with another risk-factor for COVID-19 exposure or complications (9)
- ☐ None of the above (10)

Display Question Q4.18:
If Q4.17 = I live with or care for someone with another risk-factor for COVID-19 exposure or complications

Q4.18 What other risk factors do people you live with have for COVID-19 exposure or complications?

____

Q4.19 Have you ever tested positive for COVID-19 or COVID-19 antibodies?

- ○ Yes (1)
- ○ No (2)

Q5.2 How many IGI FAST saliva tests have you had?

- ○ 0 (1)
- ○ 1 (2)
- ○ 2-4 (3)
- ○ 5-8 (4)
- ○ >8 (5)

Q5.3 Have you ever been tested for COVID-19 outside of IGI FAST (including antibody testing)?

- ○ Yes (1)
- ○ No (2)

Display Question Q5.4:
If Q5.3 = Yes

Q5.4 Where else have you had testing for COVID-19? (select all that apply)

- ☐ At a University Health Services (UHS) asymptomatic self-swab site (1)
- ☐ At the UHS Tang Center (2)
- ☐ At another site off-campus (3)

Display Question Q5.5:
If Q5.3 = Yes

Q5.5 How did your experience with IGI FAST compare to other COVID-19 testing with regards to:

	Other test(s) were better (1)	About the same (2)	IGI FAST was better (3)
Turn-around time for results (1)	○	○	○
Tolerability (2)	○	○	○
Convenience (3)	○	○	○
Professionalism (4)	○	○	○

Q5.6 How did you hear about IGI FAST? (select all that apply)

- ▢ UC Berkeley symptom screener survey response (1)
- ▢ University Health Services website (2)
- ▢ Innovative Genomics Institute website (3)
- ▢ Other UC Berkeley website (4)
- ▢ Social media (5)
- ▢ Walked by testing site (6)
- ▢ Friend/coworker (7)
- ▢ Family/household member (10)
- ▢ Email (8)
- ▢ Other (specify) (9)
- ▢ Don't know/don't remember (11)

Display Question Q5.7:
If Q5.6 = Other (specify)

Q5.7 How else did you hear about IGI FAST?

____

Q5.8 Why did you initially join IGI FAST? (select all that apply)

- ▢ I wanted to get regular testing for COVID-19 (1)
- ▢ I wanted to contribute to COVID-19 research (2)
- ▢ I was worried I was exposed to COVID-19 (3)
- ▢ I was worried I had COVID-19 symptoms (4)
- ▢ Fulfill travel requirements (10)
- ▢ Encouragement from friends/housemates/coworkers (5)
- ▢ Requirement of my employer (11)
- ▢ Pressure from a boss/supervisor (7)
- ▢ Pressure from family/friends/housemates (8)
- ▢ Other (specify) (6)

Display Question Q5.9:
If Q5.8 = Other (specify)

Q5.9 Why else did you initially join IGI FAST?

____

Display Question Q5.10:
If Q5.2 != 0
Or Q5.2 != 1

Q5.10 Why have you continued to participate in IGI FAST? (select all that apply)

- ☐ I wanted to get regular testing for COVID-19 (1)
- ☐ I wanted to contribute to COVID-19 research (2)
- ☐ It makes me feel more comfortable doing things outside of social isolation (3)
- ☐ It made me feel safer at work (4)
- ☐ Encouragement from friends/housemates/coworkers (5)
- ☐ Requirement of my employer (9)
- ☐ Pressure from a boss/supervisor (6)
- ☐ Pressure from family/friends/housemates (7)
- ☐ Other (specify) (8)

Display Question Q5.11:
If Q5.10 = Other (specify)

Q5.11 Why else did you continue to participate in IGI FAST?

____

Display Question Q5.12:
If Q5.8 = Pressure from a boss/supervisor
Or Q5.8 = Requirement of my employer
Or Q5.10 = Pressure from a boss/supervisor
Or Q5.10 = Requirement of my employer

Q5.12 Were you required or pressured to participate in IGI FAST specifically?

- ○ Yes, IGI FAST specifically (1)
- ○ No, it was my understanding that any source of COVID-19 testing would have been sufficient (2)

Q5.13 How hard was it to produce a sufficient amount of saliva for the IGI FAST test?

- ○ Extremely easy (1)
- ○ Somewhat easy (2)
- ○ Neither easy nor difficult (3)
- ○ Somewhat difficult (4)
- ○ Extremely difficult (5)

Display Question Q5.14:
If Q5.2 = 2-4
Or Q5.2 = 5-8
Or Q5.2 = >8

Q5.14 Did you develop any strategies for preparing to give a saliva sample during the study?

- ○ Yes (1)
- ○ No (2)

Display Question Q5.15:
If Q5.14 = Yes

Q5.15 What strategies did you have to help you give a saliva sample?

____
____
____
____
____

Q5.16 How well do the following terms describe your experiences with the IGI FAST test, testing sites, or personnel?

	Poor description									Excellent description
0	10	20	30	40	50	60	70	80	90	100

Easy (1)	
Tolerable (2)	
Convenient (3)	
Confusing (4)	
Safe (5)	
Professional (6)	
Respectful (7)	
Embarrassing (8)	

Q5.17 What best describes your view of the turn-around time for results with IGI FAST?

- ◯ Very slow (1)
- ◯ Slow (2)
- ◯ Neither fast or slow (3)
- ◯ Quick (4)
- ◯ Very quick (5)

Q5.18 How frequently did you travel to campus specifically to get tested for IGI FAST on a day where you would not have come to campus otherwise?

- ◯ Never (1)
- ◯ Infrequently (2)
- ◯ About half the times I got tested (3)
- ◯ Usually (4)
- ◯ Every time I got tested (5)

Display Question Q5.19:
If Q4.3 = Yes

Q5.19 How long did you need to travel from your work space to get to an IGI FAST testing site?

- ◯ 1-2 minutes (1)
- ◯ 3-5 minutes (2)
- ◯ 6-10 minutes (3)
- ◯ >10 minutes (4)

Display Question Q5.20:
If Q4.3 = No

Q5.20 How long did you typically need to travel to get to an IGI FAST testing site?

- ◯ 1-2 minutes (1)
- ◯ 3-5 minutes (2)
- ◯ 6-10 minutes (3)
- ◯ >10 minutes (4)

Display Question Q.5.21.:
If Q5.3 = Yes

Q5.21 The following table seeks to understand what other kinds of COVID-19 tests you have had. For each type of test, please indicate if you have ever had this type of test in the first column, and if you have, answer the questions in the next three columns that ask about how that test was administered and functioned.

	If yes, was it ordered by a clinician?			If yes, how long did your results take?		Did you have this kind of sample taken?		If yes, was it administered by someone else (e.g. a clinician swabbing you) or by yourself (e.g. spitting in a tube you're holding, self-swabbing)?	
	Yes (1)	No (2)	Don't know (3)	Minutes/ hours (1)	Days/weeks (2)	Yes (1)	No (2)	Took the sample myself (1)	Some one else took the sample (2)
Nasopharyngeal swab (deep in the nostril) to detect active COVID-19 infection (1)	▢	▢	▢	▢	▢	○	○	▢	▢
Nasal swab (only part way up the nostril) to detect active COVID-19 infection (16)	▢	▢	▢	▢	▢	○	○	▢	▢

Oral swab to detect active COVID-19 infection (17)	▢	▢	▢	▢	▢	◯	◯	▢	▢
Non-IGI FAST saliva to detect active COVID-19 infection (18)	▢	▢	▢	▢	▢	◯	◯	▢	▢
Blood to detect to detect active COVID-19 infection (19)	▢	▢	▢	▢	▢	◯	◯	▢	▢
Blood to detect antibodies to COVID-19 (past COVID-19 infection) (20)	▢	▢	▢	▢	▢	◯	◯	▢	▢

Q5.22 What did you find most easy or enjoyable about IGI FAST?

____

Q5.23 What did you find most difficult or frustrating about IGI FAST?

____

Q6.2 Would you continue to participate in regular (at least every two weeks) COVID-19 testing given the following scenarios? Consider each factor independently of the others.

	Yes, I'd continue to participate (1)	Maybe, I'm not sure (2)	No, I would not continue to participate (3)
It uses a self-administered nasal swab? (note: a nasal swab goes only part of the way up your nose while a nasopharyngeal swab goes all the way to the back of your upper throat) (1)	o	o	o
The only available testing sites are located at the Recreational Sports Facility (RSF) and Memorial Stadium? (2)	o	o	o
It is administered by University Health Services? (3)	o	o	o
It is administered by an external (non-UCB/IGI) provider? (4)	o	o	o
The test is clinically authorized (no confirmatory testing for positives)? (5)	o	o	o
The test is mandatory to work on campus or live in university housing? (6)	o	o	o

Q6.3 Disregarding factors like the kind of test used, would you continue participating in regular COVID-19 testing if it were recommended to be taken:

	Yes, I'd continue to participate (1)	Maybe, I'm not sure (2)	No, I would not continue to participate (3)
Once every three weeks (1)	o	o	o
Once every two weeks (2)	o	o	o
Once every week (3)	o	o	o
Twice every week (4)	o	o	o

Q6.4 What is the lowest test accuracy you would view as 'worth it' when deciding whether or not to participate in regular asymptomatic COVID-19 testing? *If you're not sure, please skip this question.*

	50	60	70	80	90	100
Test catches __% of positive cases (1)						

Q6.5 If you are exposed to COVID-19, when do you think you should first get tested? (Select all that apply)

- ☐ Same day (1)
- ☐ 1 day later (2)
- ☐ 2-3 days later (3)
- ☐ 4-6 days later (4)
- ☐ 7-9 days later (5)
- ☐ 10+ days later (6)
- ☐ Don't know (7)

Q6.6 Overall, how clear have the instructions been for selecting which COVID-19 testing options you could have used at UC Berkeley in the past three months?

- ○ Very confusing (1)
- ○ Moderately confusing (2)
- ○ Neither confusing nor clear (3)
- ○ Moderately clear (4)
- ○ Very clear (5)

Q6.7 Which of the following feelings would you anticipate having in response to a positive COVID-19 test if you were asymptomatic or mildly symptomatic at the time?

Definitely would NOT feel — Definitely would feel

0 10 20 30 40 50 60 70 80 90 100

Angry (1)	
Annoyed (2)	
Anxious (3)	
Confused (4)	
Determined (6)	
Embarrased (7)	
Grateful (8)	
Guilty (9)	
Hopeful (10)	
Like a failure (16)	
Pessimistic (11)	
Resentful (12)	
Sad (13)	
Scared (15)	

Q6.8 If you tested positive for COVID-19 right now, would you have the ability and means to self-isolate (access to private bedroom, access to private bathroom, reliable supply of food)?

- ○ Definitely (1)
- ○ Probably (2)
- ○ Maybe (3)
- ○ Probably not (4)
- ○ Definitely not (5)

Display Question Q6.9:
If Q4.3 = Yes

Q6.9 How long would you be willing to regularly travel from your workspace to get asymptomatic testing?

○ 1-2 minutes (1)

○ 3-5 minutes (2)

○ 6-10 minutes (3)

○ >10 minutes (4)

Display Question Q6.10:
If Q4.3 = Yes

Q6.10 How does your and your coworker's participation in regular asymptomatic COVID-19 testing influence your willingness to go to work?

○ I don’t feel safe working unless myself and all my coworkers to get regular COVID-19 testing (1)

○ I would prefer that myself and my coworkers to get regular COVID-19 testing (2)

○ Mine and my coworker's participation in COVID-19 testing doesn't affect how comfortable I am at work (3)

Q6.11 University Health Services is offering free regular, asymptomatic COVID-19 testing at the Recreational Sports Facility and Memorial Stadium to anyone affiliated with UC Berkeley. The test uses a self-administered nasal swab. This swab only goes part of the way up the nostril as opposed to the deep nasopharyngeal swabs you may have heard of or seen. Do you plan to continue asymptomatic COVID-19 testing through this program?

○ Yes (1)

○ Maybe (2)

○ No (3)

Q6.12 If you had the opportunity to give advice to another university setting up COVID-19 surveillance testing, what are some suggestions you would give?

____

____

____
