# Supplementary material for: Launching a saliva-based SARS-CoV-2 surveillance testing program on a university campus
Source: PLoS One. 2021 May 26;16(5):e0251296. doi: 10.1371/journal.pone.0251296 (PMC8153421; doi:10.1371/journal.pone.0251296)

**S2 Appendix**

**Visual inspection guide for on-site saliva sample screening.** See <https://innovativegenomics.org/wp-content/uploads/2021/01/visual-inspection-guide.pdf> for the full-quality version


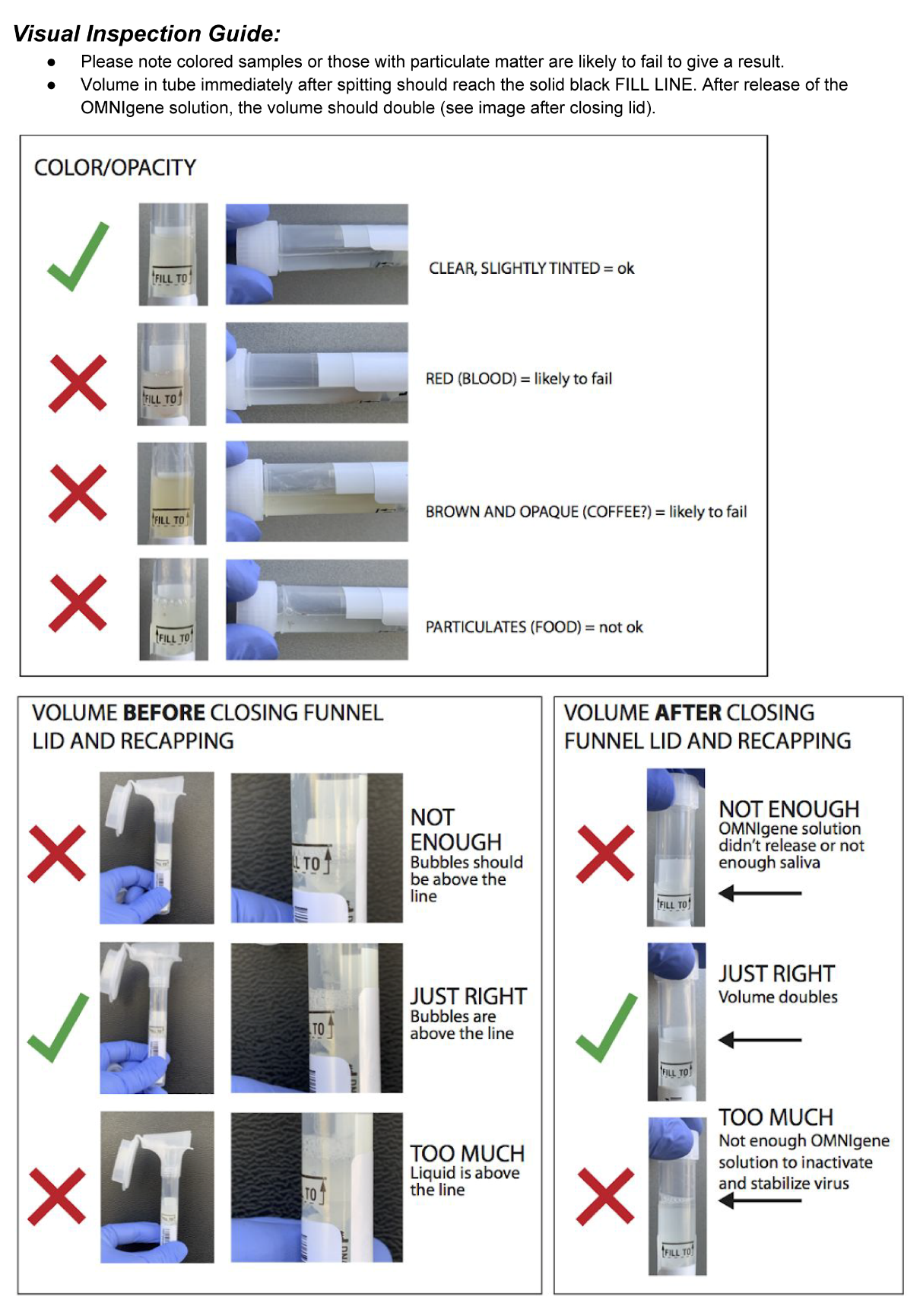

Supplement: S2 Appendix — See https://innovativegenomics.org/wp-content/uploads/2021/01/visual-inspection-guide.pdf for the full-quality version. (DOCX) [file pone.0251296.s002.docx]
