# Supplementary material for: Launching a saliva-based SARS-CoV-2 surveillance testing program on a university campus
Source: PLoS One. 2021 May 26;16(5):e0251296. doi: 10.1371/journal.pone.0251296 (PMC8153421; doi:10.1371/journal.pone.0251296)
Supplement: S4 Appendix — (DOCX) [file pone.0251296.s004.docx]

Alexandra M. Amen

Sundar Balasubramanian

Alisha Baldwin

Derek Bangs

Colin C. Barber

Lucie Bardet

Christopher Barnes

Kerrie W. Barry

John Blair

Mike Boots

John M. Boyle

Cara E. Brook

Andrew H. Doudna Cate

Seunga Choo

Alison Ciling

Maxwell Coyle

T. Cornmesser

Sumi Desai

Paige Diamond

David J. Dilworth

Mohammad Saffari Doost

Claire Dugast-Darzacq

Jennifer A. Doudna

Alexander J. Ehrenberg

David C. Ensminger

Indro Fedrigo

Phillip A. Frankino

Skyler E. Friedline

Chris Gawronski

Kristina Geiger

Petros Giannikopoulos

Holly K. Gildea

Thomas G. W. Graham

Ralph Green

Michael Hunter Green

Allan Gopez

Jennifer R. Hamilton

Anna Harte

Stephanie G. Hays

Ariana Hirsh

Lauren Hunter

Megan L. Hochstrasser

Dirk Hockemeyer

Carina Jaegers

Nicholas Karavolias

Jonathan Karr

Amanda Keller

Netravathi Krishnappa

Jamie Lahvic

Azra Lari

Emily K. Lam

Siyoun Lee

Hanqin Li

Kaitlyn N. Letourneau

Enrique Lin-Shiao

Franziska Lorbeer

Tianlin Lu

Mariya Lukarska

Karen Lundy

Ana Lyons

Elijah F. Lyons

Antonio Maffia

Anna Maurer

Kevin G. Mark

Lisa Argento Martell

Raquel O. Martins

Shally Margolis

Kevin G. Mark

Anna Maurer

Shana L. McDevitt

Matthew McElroy

Riley McGarrigle

Alina Minikhanova

Patrick S. Mitchell

Erica A. Moehle

Andrew J. Modzelewski

Andrew Murley

Andrew G. Murdock

Christine Naca

Divya Nandakumar

Ben Natan

Guy Nicolette

Rebekah Oakes

Elizabeth O’Brien

Derek J. Pappas

Janneke Peeters

Maya Petersen

Corinne Pender

Kathleen Pestal

Kaila Pianalto

Diana L. Quach

Bradley R. Ringeisen

Gabriela Rios-Sotelo

Benjamin E. Rubin

Rohan Sachdeva

Catherine Schneider

Carolyn Sherry

Korey Sop

Michael Stadler

Elizabeth C. Stahl

Abdullah Muhammad Syed

Hailey Stynes

Iman Sylvian

I-Li Tan

Janina Tamborski

Amy L. Tollner

Connor A. Tsuchida

Kimberly Tsui

Alexandra Tsitsiklis

Timothy K. Turkalo

Fyodor D. Urnov

Bryan Warf

Oscar N. Whitney

Lea B. Witkowsky

Luis Valentin-Alvarado

Clara Williams

Kurtresha Worden

Gabrielle Wright

Madeleine Zhu

Arielle Zur
